# Geochemical and mineralogical data of wildfire ash and soil samples from post-fire areas: A case study of the August 2024 wildfire in Attica, Greece

**DOI:** 10.1016/j.dib.2026.112608

**Published:** 2026-02-19

**Authors:** Triantafyllia Samara, Panagiotis Papazotos, Alexandros Liakopoulos, Marina Karaevangelou, Elissavet-Antonia Georgolopoulou, Vassiliki Angelatou, Dimitrios Tarenidis

**Affiliations:** Hellenic Survey of Geology and Mineral Exploration, 1 Sp. Louis str., 13677 Acharnes, Greece

**Keywords:** PTEs, PAHs, Soil geochemistry, Geo-environmental indices, Wildfire-induced changes, Metal(oid)s, Soil ecotoxicology, XRD

## Abstract

This data article provides supplementary information to “Delineation of wildfire impacts on soil geochemistry and post-fire soil quality assessment: The case of the August 2024 wildfire in NE Attica, Greece” [1]. An extended dataset records the soil geochemical impacts of the August 2024 Attica wildfire, supporting post-fire soil quality and environmental geochemistry assessment. Raw geochemical data, including pH, major oxides, Polycyclic Aromatic Hydrocarbons (PAHs), Major Elements (MEs), Potentially Toxic Elements (PTEs) and other Trace Elements (TEs), are presented, along with X-ray diffraction (XRD) patterns, and three widely used geo-environmental indices, such as Enrichment Factor (EF), Pollution Load Index (PLI), and Geoaccumulation Index (I_geo_) from post-fire samples. Wildfire Ash (WA) (depth: 0-2 cm) and soil (Burned [BS] and unburned [NB]) Samples (depth: 2-20 cm) were collected in a sampling campaign carried out during October 2024. This dataset is useful to evaluate the immediate post-fire geochemical impacts by providing a large set of geochemical soil analyses data across 52 samples, addressing knowledge gaps in wildfire-driven contaminant cycling in Mediterranean soils, supporting environmental risk assessment and post-fire management, and establishing a baseline for long-term monitoring and reproducible research. Herein, the methodological limitations related to sampling design, temporal coverage, analytical scope, and baseline data availability are explicitly acknowledged, ensuring transparency in the data interpretation and providing guidance for future research.

Specifications Table

**Table:** 

Subject	Earth & Environmental Sciences
Specific subject area	Soil Geochemistry, Environmental Science.
Type of data	Table, Figure, Raw data, Processed data, XRD data
Data collection	Samples were collected during a sampling campaign conducted in October 2024. Pre-treatment for chemical determinations included air-drying at 40°C, gentle disaggregation, homogenization, sieving to < 2 mm, and fine grinding to < 0.075 mm. Chemical analyses were carried out using ICP-AES and ICP-MS following aqua regia digestion, while major oxides were determined by XRF using the fusion method in an EQUILAB FX Induction Fluxer, employing lithium tetraborate (Li_2_B_4_O_7_) as flux for sample preparation. An automated Bruker AXS S4 Pioneer spectrometer was employed. PAHs were measured by GC-MS after hexane extraction, and pH was determined in water and 0.01 M KCl suspensions using a calibrated pH meter. Mineralogical characterization was performed by XRD using a PANalytical X’Pert PRO diffractometer (2θ range and scan rate: 2θ=3-70°, 0.02°/2s), operating at a current of 40 kV and 30 mA. Phase identification and semi-quantitative mineralogical analysis were carried out using PANalytical X’Pert HighScore software by Malvern Panalytical, with an estimated DL≈ 1 wt%. Secondary data, such as geo-environmental indices were calculated using Microsoft Excel.
Data source location	Location: NE Attica, Greece Country: Greece Geographical boundaries of the survey area in the World Geodetic System 1984 (WGS84) coordinate reference system: Northwest (NW) boundary (Latitude/Longitude): 38°15′12″ N / 23°50′28″ E Northeast (NE) boundary (Latitude/Longitude): 38°15′12″ N / 23°57′58″ E Southeast (SE) boundary (Latitude/Longitude): 38°01′00″ N / 23°57′58″ E Southwest (SW) boundary (Latitude/Longitude): 38°01′00″ N / 23°50′28″ E
Data accessibility	The dataset, which includes raw chemical analysis data, secondary geo-environmental index data, XRD raw files, and XRD figures, is hosted by: Repository name: Mendeley Data repository DOI: 10.17632/c688m2wpkp.1 Direct link to the dataset: https://data.mendeley.com/datasets/c688m2wpkp/1
Related research article	Author’s name Papazotos, P., Samara, T., and Liakopoulos, A. Title Delineation of wildfire impacts on soil geochemistry and post-fire soil quality assessment: The case of the August 2024 wildfire in NE Attica, Greece Journal Applied Geochemistry

## 1 Value of the Data

• The dataset provides a high-resolution geochemical profile of the post-fire impact of the August 2024 wildfire in Attica, Greece, by comprehensively characterizing a large set of chemical elements and other compounds, including Major Elements (MEs), Potentially Toxic Elements (PTEs), Trace Elements (TEs), and Polycyclic Aromatic Hydrocarbons (PAHs), across 52 Wildfire Ash (WA) and soil samples.
• The dataset addresses key scientific gaps in understanding how fire events affect the occurrence, mobility, and distribution of contaminants in Mediterranean soils, providing data that can improve understanding of post-fire contaminant cycling.
• The dataset has strong regional relevance, as its findings are specific to the geochemical, pedological, and environmental conditions of the Mediterranean region, making it invaluable for comparison and modeling studies in similar worldwide fire-prone ecosystems.
• The data offers detailed combined geochemical and mineralogical information on WA and soils affected by the August 2024 Attica wildfire, enabling geochemists, mineralogists, environmental scientists, agronomists, engineers, and public health specialists to quantify contaminant contents, track their post-fire mobility, and identify soil recovery patterns, directly supporting environmental risk assessments and remediation strategies.
• The dataset establishes a baseline of 52 well-characterized samples, allowing future research to monitor contaminant evolution over time, assess the soil's natural or engineered recovery rate, and improve predictive models of post-fire soil geochemistry.
• The data adds significant supplementary information to the associated research article [1], greatly promoting transparency and reproducibility by making the validated data publicly available, allowing other researchers to replicate analyses, validate interpretations, and conduct secondary data analyses.

## 2 Background

The theoretical motivation for this dataset lies in the critical need to understand the immediate impact of climate-driven wildfires on soil geochemistry. Despite known general alterations, the specific effects on PTE and PAH environmental fate, and the mechanisms governing their mobilization in Mediterranean soils remain poorly characterized. Therefore, geochemical, mineralogical, and field data are important tools for investigating the origin, occurrence, transport, and fate of these substances in fire-prone environments. On 11^th^ August 2024, a major wildfire incident occurred in NE Attica, Greece. The fire originated in the broader area of Varnavas Town, about 32 km northeast of central Athens, and quickly advanced southward along three primary fronts. It affected several settlements and areas, including Grammatiko, Marathon, Nea Makri, and Mount (Mt.) Penteli, before reaching the northern suburbs of Athens, such as Vrilissia and Chalandri. This incident provided a unique opportunity to investigate the geochemical and mineralogical changes across a case in the Mediterranean region via a systematic soil sampling campaign. Methodologically, this data article aims to support a combined assessment of post-fire soil geochemistry and mineralogy, using the August 2024 Attica wildfire as an ideal example. The dataset was compiled following the August 2024 wildfire in Attica, Greece, to capture a detailed, high-resolution geochemical profile using a systematic sampling grid and advanced analytical techniques. This data article adds significant value to our original research publication titled “Delineation of wildfire impacts on soil geochemistry and post-fire soil quality assessment: The case of the August 2024 wildfire in NE Attica, Greece” [1] offers additional value by providing the complete, validated dataset for 52 collected samples, containing WA, Burned Soils (BS), unburned (Not Burned) soils (NB), and field blanks. Furthermore, additional Quality Control (QC) data are provided, including two laboratory blanks, and two Standard Reference Materials (SRMs). The dataset is complemented with X-ray fluorescence (XRF) analyses and X-ray diffraction (XRD) patterns of selected WA and BS samples from representative sites, chosen based on spatial distribution, geological features, land use, and the contents of the most environmentally and toxicologically significant PTEs. This transparency is important for validating our findings and establishing a reliable, open-access baseline for future long-term environmental studies, predictive modeling, and secondary analyses by the broader scientific community, policymakers, and other stakeholders.

## 3 Data Description

**Fig. 1:**
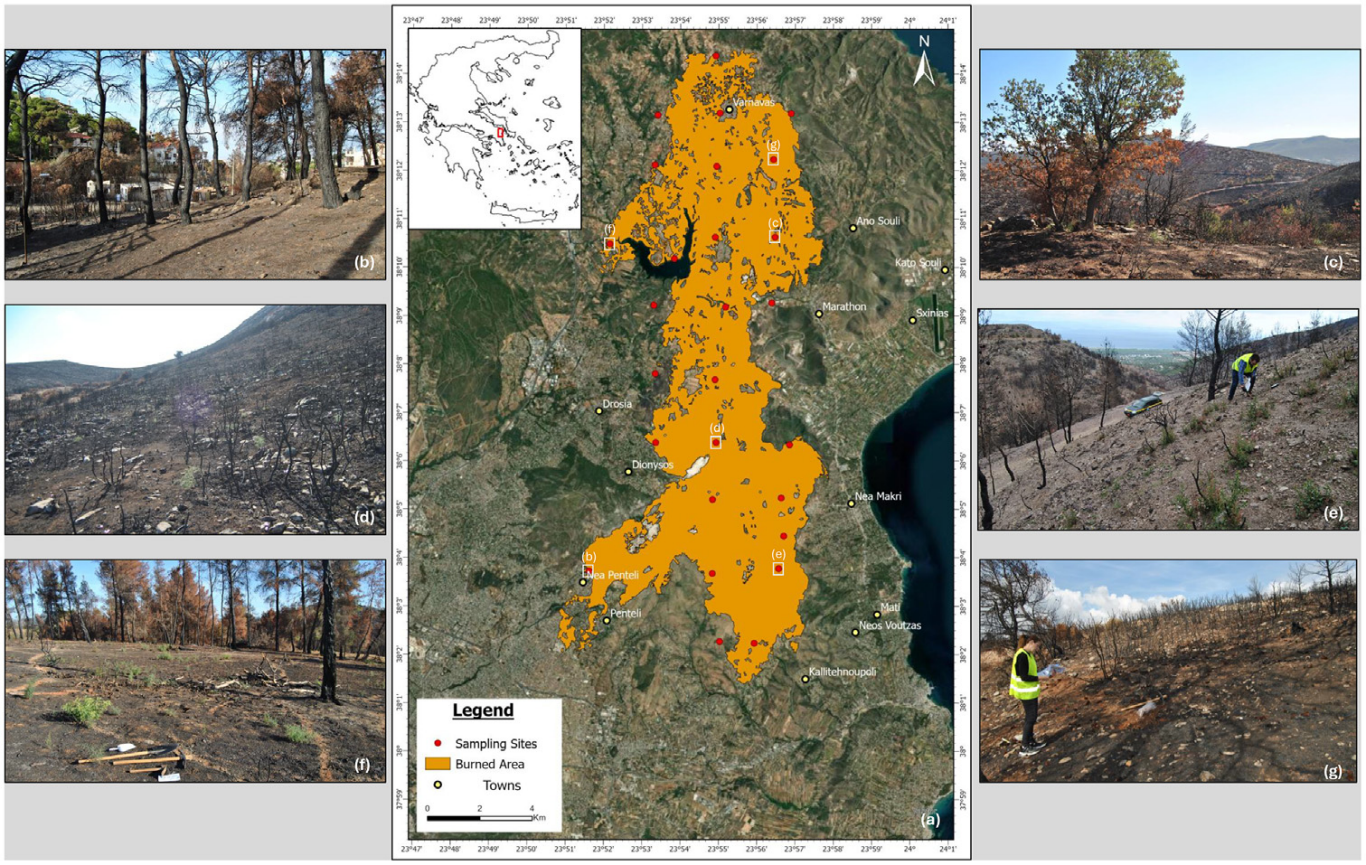
Map showing the sampling locations (a) and representative photographs of the sampling activities and sites (b-g); white squares indicate the locations of the photographs.

A total of 52 samples were collected from 27 distinct sites during a sampling campaign carried out in October 2024 (Fig. 1a).

Regarding sampling, a total of 32 soil samples (26 BS and six NB), 18 WA samples, and two field blanks, including duplicate samples were collected. To ensure data quality and representativeness, seven duplicate samples were randomly selected, including three BS and two WA samples. The datasets are presented on four Excel files as described below:

The *Sites.xlsx file* has four columns that represent:

• Sample Identification Code (ID).
• Type of the sample: WA samples, soil (BS and NB) samples, Field and Lab Blanks samples and SRMs.
• Sampling site locations referenced to the World Geodetic System 1984 (WGS84) coordinate reference system.
• Code analysis was conducted using a randomized sample order submitted by Hellenic Survey of Geology & Mineral Exploration (HSGME) research team to the SGS analytical laboratory to ensure QC and data reliability.

The *GeochAnalysis1.xlsx file* contains content data of 51 MEs, PTEs, and TEs and 10 major oxides:

• Al, Ca, Fe, K, Mg, Na, P, S, and Ti contents expressed as weight percentages (wt%).
• Ba, Cr, Cu, Li, Mn, Sr, V, Zn, Zr, Ag, As, Be, Bi, Cd, Ce, Co, Cs, Ga, Ge, Hf, Hg, In, La, Lu, Mo, Nb, Ni, Pb, Rb, Sb, Sc, Se, Sn, Ta, Tb, Te, Th, TI, U, W, Y, and Yb contents expressed as mg/kg.
• Major oxide (SiO_2_, Al_2_O_3_, Fe_2_O_3_, MnO, MgO, CaO, Na_2_O, K_2_O, TiO_2_, P_2_O_5_) contents and Loss On Ignition (LOI) were determined for 10 representative WA and BS samples and are reported as oxide weight percentages normalized to 100% (SUM+LOI). A comparison between measured and expected values for international XRF reference standards (major oxides, wt% normalized to 100) is also provided, with certified values highlighted in green.

The *GeochAnalysis2.xlsx file* includes pH values and content data for 40 selected BS and WA samples. A total of 16 PAHs (Naphthalene [Nap], Acenaphthylene [Acy], Acenaphthene [Ace], Fluorene [Flu], Phenanthrene [Phe], Anthracene [Ant], Fluoranthene [Flt], Pyrene [Pyr], Benz(a)anthracene [BaA], Chrysene [Chr], Benzo(b)fluoranthene [BbF], Benzo(k)fluoranthene [BkF], Benzo(a)pyrene [BaP], dibenzo(a,h)anthracene [DaA], Benzo(g,h,i)perylene [BgP], and Indeno(1,2,3-c,d)pyrene [Ind]) were determined. Furthermore, Total PAHs (TPAH) content is provided. All PAH contents expressed as mg/kg. Additionally, samples for which analyses were not conducted are marked as Not Available (NA) in the provided file.

The *Geo-environmental_Indices.xlsx file* records the calculations of data processing for three widely used geo-environmental indices: the Enrichment Factor (EF), Pollution Load Index (PLI), and Geoaccumulation Index (I_geo_). These indices were applied to assess PTEs loads using metal(loid)s of major toxicological and environmental significance (i.e., Cr, Cu, Zn, As, Cd, Hg, Ni, Pb, Sb, Sn, and U) [2]. These secondary data provide a robust evaluation of contamination levels and associated environmental impacts, offering valuable insight into post-fire soil quality and contamination status.

Furthermore, X-ray diffraction (XRD) patterns of the ten analyzed WA and BS samples, along with their semi-quantitative mineralogical compositions, are provided.

## 4 Materials and Methods

### 4.1 Soil sampling procedure

**Fig. 2:**
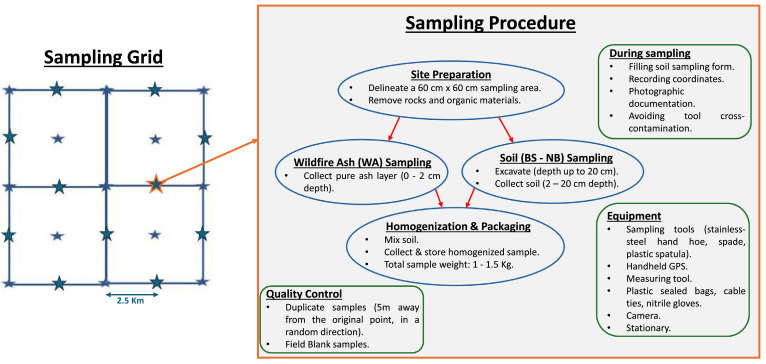
Sampling grid and workflow of the sampling procedure.

The sampling procedure was carried out using a grid with a cell size of 2,500 m × 2,500 m, covering an area of 104 km^2^, following the grid system of the Geochemical Atlas of Greece (GAG), which features a cell size of 5,000 m × 5,000 m, in accordance with the sampling procedure established by the Department of Geochemistry and Environment of the Hellenic Survey of Geology and Mineral Exploration (HSGME) [3]. This sampling procedure includes methodological principles and specifications outlined by the EuroGeoSurveys Geochemistry Group [4] and the International Union of Geological Sciences (IUGS) / Commission on Global Geochemical Baselines (CGGB) [4]. In addition to the primary grid of GAG [3], supplementary sampling points were established at the center of each grid cell (Fig. 2) and at the midpoints of the sides of the grid cells. Subsequently, this sampling design was implemented within the polygon delineating the boundaries of the area of interest to densify the sampling network and ensure the collection of a sufficient number of samples to assess the impact of the wildfire of 11^th^ August 2024 on soil quality. This sampling strategy offers several advantages: (a) uniform and comprehensive spatial coverage; (b) flexibility to further densify the grid; (c) ability to identify systematic spatial trends in the contents of chemical elements; and (d) straightforward design and rapid field implementation. Despite these advantages, a limitation of this approach is the reduced likelihood of detecting geochemical anomalies with spatial extents smaller than the grid cell dimensions. This limitation is mitigated by the collection of samples from the four vertices of each grid cell in combination with its central point.

**Fig. 3:**
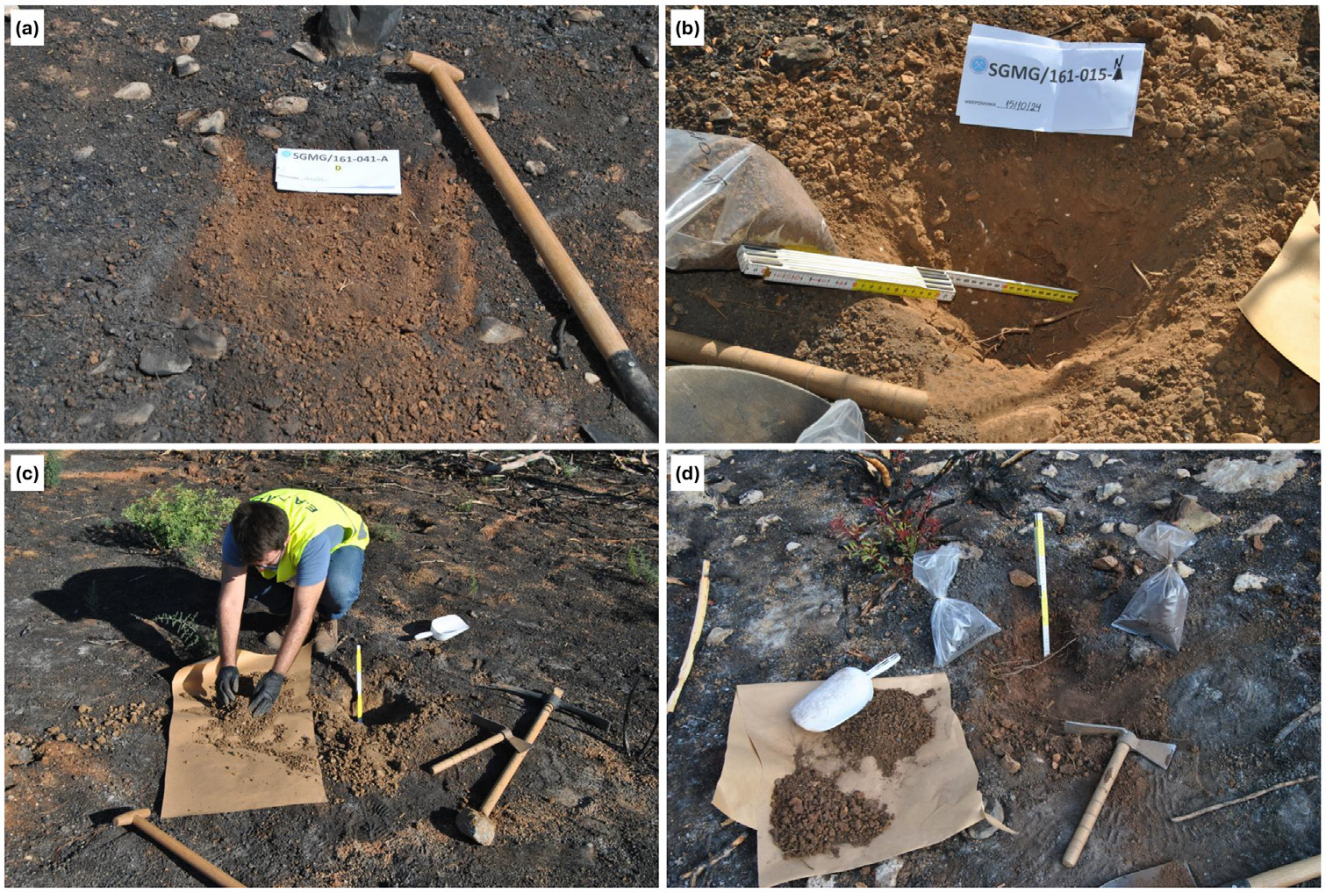
Representative photographs illustrating the sampling stages, including field site conditions, collection of samples, sample handling, and packaging for analysis (a-d).
• A clean square area measuring 60 × 60 cm was delineated.
• A card displaying the sampling ID was placed within the delineated area.
• Photographs were taken, including general and close-up views of the sampling area, to document the site's main characteristics.
• Sampling tools were preconditioned with soil from the site to minimize cross-contamination.
• The upper 20 cm of surface soil was excavated using sampling tools, such as a stainless-steel hand hoe and spade.
• The excavated soil (NB and BS) was homogenized at the sampling site using disposable nitrile gloves to obtain a representative bulk sample from the 0–20 cm depth interval (Figs. 3b-3c).
• The homogenized soil samples were placed in plastic bags and sealed airtight using cable ties.
• Each sample was labeled with its predetermined ID on a sampling tag and on the exterior of the sampling bag. The sealed bags were placed in cardboard boxes for safe transport to the laboratory. Each sample weighed approximately 1–1.5 kg (Fig. 3d).
• The exact geographic location of each sampling point was determined using a handheld Global Positioning System (GPS) device (GPSMAP 276Cx), and coordinates were recorded in the WGS84 reference system.
• Duplicate samples were collected using the same procedure about 5 m from the primary sampling point, in a random direction, using the same rigorous sampling procedure; duplicate samples constitute ≈ 10% of the total sampling sites. This distance was selected to account for potential errors in estimating the precise sampling location and corresponded to the positional deviation indicated by the GPS, thereby supporting data accuracy and reliability. Additionally, field blanks were included in the sampling procedure to assess potential contamination during sampling; these blanks were collected using the same procedure and equipment described above.
• After each sampling, all tools were thoroughly cleaned to prevent cross-contamination during later use.
• Photographic documentation of the sampling location was obtained during soil collection.

All samples collected for the first time were assigned a unique ID based on the map sheet (i.e., the map number provided by the Hellenic Military Geographical Service [HMGS]) and the sample serial number. A standardized soil sampling form was developed and completed in situ for each sample. The soil sampling procedure followed the steps outlined below, as summarized in Fig. 2:

• Before sampling, all relevant information was recorded on the soil sampling form, including key site observations such as land use, soil color, soil category and subcategory, topography, geology, and vegetation cover.
• Before sample collection, the sampling point was superficially cleaned by removing rocks and organic material (e.g., herbaceous vegetation, grass, organic residues, etc.). It is noted that WA samples were collected from the pure ash layer (0–2 cm) (Fig. 3a).

Limited accessibility and sampling difficulties at some sites meant that it was not always possible to reach the planned sampling locations with absolute precision. Minor deviations between the initial and final sampling locations were unavoidable. It is stated that all samples were collected at least 200 meters from main roads, rubble, power poles, anthropogenic interventions, and other potential local sources of pollution.

### 4.2 Sample preparation & pre-treatment

The sample preparation and pre-treatment for chemical analyses occurred at the HSGME. To preserve the integrity of the samples, particularly regarding volatile elements, they were oven dried at a stable temperature of 40°C. Dry samples were gently disaggregated in a mortar with a ceramic pestle, then thoroughly mixed to ensure uniformity. The material was passed through a nylon sieve < 2 mm to remove larger particles. After sieving, the samples were finely ground using an agate ball mill until a grain size < 0.075 mm was achieved.

### 4.3 Geochemical analyses

All samples were analyzed using Inductively Coupled Plasma Atomic Emission Spectroscopy (ICP-AES) and Inductively Coupled Plasma Mass Spectrometry (ICP-MS) at SGS Analytical Laboratories in Canada, and a total of 61 chemical elements, including major oxides, PTEs, MEs, and TEs, were determined. It is noted that MEs (e.g., Al, Fe, Ca, Mg, etc.), constitute the primary components of soil and are typically expressed as % (Table 1). In contrast, TEs (e.g., Rare Earth Elements - REEs, Ba, Zr, V, etc.) are present at lower contents and are usually reported in mg/kg (Table 1). Elements that can pose health risks to living organisms, even at low contents, are classified as PTEs (e.g., As, Cd, Cr, Pb, Zn, etc.) [2]. The analytical procedure involved aqua regia digestion using a 3:1 mixture of HCl and HNO_3_. This digestion was performed at 95°C for 1 hour on <10 g of finely milled sample material with particle size <0.075 mm, following the protocol outlined by Reimann et al. [5]. The PAH content and pH of 40 selected BS and WA samples were analyzed at SGS laboratories in Germany. PAHs were quantified using Gas Chromatography–Mass Spectrometry (GC-MS) following hexane extraction (DIN ISO 18287). The pH was measured in both water and 0.01 M KCl suspensions using a calibrated pH meter (DIN EN 12176). All geochemical analyses were carried out according to the ISO/IEC 17025:2005 standard, ensuring compliance with internationally recognized protocols for Quality Assurance (QA) and QC. The Detection Limits (DLs) and Upper Limits (ULs) of the ICP-AES/MS and GC-MS for each chemical compound are given in Table 1.

**Table 1:** DLs and ULs of the analytical methods used for each chemical element in the geochemical research on the August 2024 wildfire in Attica, Greece.

Element or chemical compound	Method	Unit	DL	UL
Al	ICP-AES	%	0.01	15
Ba	ICP-AES	mg/kg	5	10000
Ca	ICP-AES	%	0.01	15
Cr	ICP-AES	mg/kg	1	10000
Cu	ICP-AES	mg/kg	0.5	10000
Fe	ICP-AES	%	0.01	15
K	ICP-AES	%	0.01	15
Li	ICP-AES	mg/kg	1	10000
Mg	ICP-AES	%	0.01	15
Mn	ICP-AES	mg/kg	2	10000
Na	ICP-AES	%	0.01	15
P	ICP-AES	%	0.01	15
S	ICP-AES	%	0.01	5
Sr	ICP-AES	mg/kg	0.5	10000
Ti	ICP-AES	%	0.01	15
V	ICP-AES	mg/kg	1	10000
Zn	ICP-AES	mg/kg	1	10000
Zr	ICP-AES	mg/kg	0.5	10000
Ag	ICP-MS	mg/kg	0.01	100
As	ICP-MS	mg/kg	1	10000
Be	ICP-MS	mg/kg	0.1	100
Bi	ICP-MS	mg/kg	0.02	10000
Cd	ICP-MS	mg/kg	0.01	10000
Ce	ICP-MS	mg/kg	0.05	1000
Co	ICP-MS	mg/kg	0.1	10000
Cs	ICP-MS	mg/kg	0.05	1000
Ga	ICP-MS	mg/kg	0.1	10000
Ge	ICP-MS	mg/kg	0.1	10000
Hf	ICP-MS	mg/kg	0.05	500
Hg	ICP-MS	mg/kg	0.01	100
In	ICP-MS	mg/kg	0.02	500
La	ICP-MS	mg/kg	0.1	10000
Lu	ICP-MS	mg/kg	0.01	1000
Mo	ICP-MS	mg/kg	0.05	10000
Nb	ICP-MS	mg/kg	0.05	1000
Ni	ICP-MS	mg/kg	0.5	10000
Pb	ICP-MS	mg/kg	0.2	10000
Rb	ICP-MS	mg/kg	0.2	10000
Sb	ICP-MS	mg/kg	0.05	10000
Sc	ICP-MS	mg/kg	0.1	10000
Se	ICP-MS	mg/kg	1	1000
Sn	ICP-MS	mg/kg	0.3	1000
Ta	ICP-MS	mg/kg	0.05	10000
Tb	ICP-MS	mg/kg	0.02	10000
Te	ICP-MS	mg/kg	0.05	1000
Th	ICP-MS	mg/kg	0.1	10000
Tl	ICP-MS	mg/kg	0.02	10000
U	ICP-MS	mg/kg	0.05	10000
W	ICP-MS	mg/kg	0.1	10000
Y	ICP-MS	mg/kg	0.05	10000
Yb	ICP-MS	mg/kg	0.1	100
Nap	GC-MS	mg/kg	0.05	-
Acy	GC-MS	mg/kg	0.05	-
Ace	GC-MS	mg/kg	0.05	-
Flu	GC-MS	mg/kg	0.05	-
Phe	GC-MS	mg/kg	0.05	-
Ant	GC-MS	mg/kg	0.05	-
Flt	GC-MS	mg/kg	0.05	-
Pyr	GC-MS	mg/kg	0.05	-
BaA	GC-MS	mg/kg	0.05	-
Chr	GC-MS	mg/kg	0.05	-
BbF	GC-MS	mg/kg	0.05	-
BkF	GC-MS	mg/kg	0.05	-
BaP	GC-MS	mg/kg	0.05	-
DaA	GC-MS	mg/kg	0.05	-
BgP	GC-MS	mg/kg	0.05	-
Ind	GC-MS	mg/kg	0.05	-

A total of ten representative WA and BS samples were prepared for XRF analysis. Sample preparation for major oxides analysis was carried out using the fusion method in an EQUILAB FX Induction Fluxer, employing lithium tetraborate (Li_2_B_4_O_7_) as flux. Elemental analysis for the determination of major oxides (i.e., SiO_2_, Al_2_O_3_, Fe_2_O_3_, MnO, MgO, CaO, Na_2_O, K_2_O, TiO_2_, P_2_O_5_ - expressed as oxide wt%) was performed using XRF spectrometry on an automated Bruker AXS S4 Pioneer spectrometer. Loss on ignition (LOI) was determined after heating at 1000°C for 2 h. Quality Control (QC) of the elemental analysis was ensured through multiple measurements and replicate analyses of selected samples to assess measurement repeatability.

### 4.4 X-ray diffraction (XRD)

X-ray diffraction (XRD) analyses were performed using a PANalytical X’Pert PRO diffractometer with Cu radiation (40 kV, 30 mA). Data were collected over a 2θ range of 3°–70° with a step size of 0.02° and a counting time of 2 s per step under continuous scanning, at 25°C with sample spinning. Phase identification and semi-quantitative mineralogical analysis were carried out using the PANalytical X’Pert HighScore software by Malvern Panalytical, with an estimated DL of approximately 1 wt%. XRD patterns of the ten analyzed WA and BS samples, together with their semi-quantitative mineralogical composition, are presented in Figures S1-S10. Table 2 summarizes the identified minerals, classified by mineralogical group, and their frequency of occurrence in the examined samples.

**Table 2:** Frequency of occurrence of minerals identified by XRD in the analyzed samples.

				CODE									
MINERAL GROUPS		MINERALS	INDICATIVE CHEMICAL FORMULA	161-026A	161-014A	161-025A	161-033A	161-005A	161-026B	161-014B	161-025B	161-033B	161-005B
SILICATES		Quartz	SiO₂	Χ	Χ	Χ	Χ	Χ	X	X	X	X	Χ
		Plagioclase	(Na,Ca)(Si,Al)₄O₈	Χ	Χ	Χ	Χ	Χ	X	X	X	X	Χ
		K-feldspar	KAlSi₃O₈	Χ									
		Amphibole group	(Ca,Na)₂(Mg,Fe,Al)₅(Si,Al)₈O₂₂(OH)₂	Χ								Χ	
	CLAY MINERALS	Kaolinite	Al₂Si₂O₅(OH)₄	Χ		Χ	Χ	Χ					
		Montmorillonite	(Al,Mg)₂Si₄O₁₀(OH)₂·nH₂O	Χ			Χ	Χ	X				
		Muscovite / Illite	KAl₂(AlSi₃)O₁₀(OH)₂	Χ	Χ	Χ	Χ	Χ	X	X	X	X	Χ
		Chlorite group	(Mg,Fe,Al)₆(Si,Al)₄O₁₀(OH)₈	Χ			Χ	Χ	X	X	X	X	Χ
CARBONATES		Calcite	CaCO₃	Χ		Χ	Χ	Χ	X			X	Χ
		Dolomite	CaMg(CO₃)₂		Χ		Χ		X				
SULFATES		Sodium sulfate	Na₂SO₄		Χ								
ZEOLITES		Zeolite group	(Ca,Na,K)_x_Al_x_Si_1-x_O₂·nH₂O									Χ	
OXIDES		Hematite	Fe₂O₃										Χ

### 4.5 Quality control (QC)

The QC of the analytical data quality was achieved through the following procedures:

• A total of four blank samples were analyzed: two field blanks (representing soil sample handling in the field) and two laboratory blanks (representing laboratory procedures). Data are provided in the GeochAnalysis1.xlsx file.
• Two SRMs, OREAS 502c and OREAS 151b, were analyzed to assess accuracy and precision (data provided in GeochAnalysis1.xlsx file).
• Seven duplicate samples were analyzed in random order within the overall sample sequence: four BS and three WA samples. Data are provided in the GeochAnalysis1.xlsx file.
• Considering XRF analyses, the accuracy of elemental analysis using fused beads is demonstrated by the close agreement between measured and certified values of international reference standards analyzed under the same conditions as the studied soil samples (Data are provided in the GeochAnalysis1.xlsx file). Certified values are highlighted in green color. The selected standards approximately cover the compositional range of the investigated samples.

### 4.6 Geo-environmental indices

A total of three geo-environmental indices, Geoaccumulation Index (I_geo_), Enrichment Factor (EF), and Pollution Load Index (PLI), were calculated based on the equations presented in Table 3. Definitions and interpretative frameworks are also included. Data are provided in the *Geo-environmental_Indices.xlsx file*.

**Table 3:** Equations, definitions, and interpretative framework of geo-environmental indices (Igeo, EF, and PLI).

Geo-environmental index	Equation	Definitions	Interpretative framework
Geoaccumulation Index (I_geo_)	$I_{geo}=log_{2}\left[\frac{C_{n}}{\left(1.5\times C_{bn}\right)}\right]$	C_n_: content of a PTE in the soil C_bn_: geochemical background level based on the average composition of the Earth’s crust using [6] factor 1.5: included to correct for lithogenic variations	I_geo_ < 0: no pollution I_geo_ = 0–1: low pollution I_geo_ = 1–2: moderate pollution I_geo_ = 2–3: moderate to high pollution I_geo_ = 3–4: high pollution I_geo_ = 4–5: high to very high pollution I_geo_ > 5: extremely high pollution [7]
Enrichment Factor (EF)	$EF=\frac{{\left(\frac{El}{Fe}\right)}_{Sd}}{{\left(\frac{El}{Fe}\right)}_{Bg}}$	$\frac{El}{Fe}$: a ratio of Element/Fe in the soil $\frac{El}{Fe}$: a ratio of Element/Fe in the background	EF < 2: minimal enrichment EF = 2–5: moderate enrichment EF = 5–20: significant enrichment EF = 20–40: very high enrichment EF > 40: extremely high enrichment [8]
Pollution Load Index (PLI)	$PLI={\left(CF1\times CF2\times \ldots \;\times CFn\right)}^{\frac{1}{n}}$	n: total number of PTEs/TEs CF: contamination factor of each PTE/TE	PLI < 1: unpolluted conditions; PLI = 1-2: low to moderately polluted PLI = 2-3: moderately polluted PLI = 3-4: moderately to highly polluted PLI = 4-5: highly polluted PLI > 5: signify very highly polluted [9,10]

## Limitations

The limitations of this dataset are explicitly acknowledged, and these factors inform future research priorities. Comparability among sample types at the same site was constrained by the limited number of shared samples. The lack of previous geochemical baseline studies in the region highlights the importance of developing a Geochemical Atlas of Greece (GAG). The presence of different geological formations and varying land uses contributes to significant spatial variability in elemental distributions. Sampling was conducted approximately two months after the wildfire, indicating that element contents are sensitive to the timing of sampling relative to the wildfire incident. As dataset is based on a single campaign conducted in October 2024, spatiotemporal interpretations are limited, and repeated sampling closer to the incident and at regular intervals thereafter would be required to robustly assess post-fire spatiotemporal variability in elemental occurrence. Furthermore, pH, PAHs, XRD patterns, and XRF analyses were conducted only on selected samples; 40 samples for pH and PAHs, and 10 samples for XRD and XRF. High DLs, low ULs for certain compounds, and a limited number of duplicate samples reduce analytical precision and robustness. Nevertheless, transparent identification of these limitations enhances the data interpretation and establishes a clear framework for future, more comprehensive investigations.

## Ethics Statement

This work did not involve human subjects, animal experiments, or any data collected from social media platforms.

## CRediT Author Statement

**Triantafyllia Samara:** Conceptualization, Methodology, Formal analysis, Software, Data curation, Writing – original draft, Visualization, Investigation, Validation, Fieldwork; **Panagiotis Papazotos:** Conceptualization, Methodology, Formal analysis, Software, Data curation, Writing – original draft, Visualization, Investigation, Supervision, Geochemical analysis, Fieldwork; **Alexandros Liakopoulos:** Conceptualization, Methodology, Data curation, Investigation, Supervision, Validation, Resources, Project administration, Funding acquisition, Writing – review and editing, Fieldwork; **Marina Karaevangelou:** Methodology, Software, Writing – review and editing, XRD and XRF analysis; **Elissavet-Antonia Georgolopoulou:** Methodology, Writing – review and editing, Sample preparation; **Vassiliki Angelatou:** Methodology, Writing – review and editing, Sample preparation; **Dimitrios Tarenidis:** Methodology, Writing – review and editing, XRD and XRF analysis.

## Declaration of Generative AI and AI-Assisted Technologies in the Manuscript Preparation Process

During the preparation of this work the author(s) used ChatGPT (OpenAI) in order to assist with language refinement, and clarity of academic writing. After using this tool/service, the author(s) reviewed and edited the content as needed and take(s) full responsibility for the content of the published article.

## Data Availability

Mendeley DataGeochemical and mineralogical dataset of post-fire soils (Attica, Greece) (Original data)

## Appendix Supplementary materials

Image, application 1
