# Supplementary material for: Geochemical and mineralogical data of wildfire ash and soil samples from post-fire areas: A case study of the August 2024 wildfire in Attica, Greece
Source: Data Brief. 2026 Feb 19;65:112608. doi: 10.1016/j.dib.2026.112608 (PMC12966705; doi:10.1016/j.dib.2026.112608)
Supplement: Supplementary file 1 [file mmc1.pdf]

Supplementary material

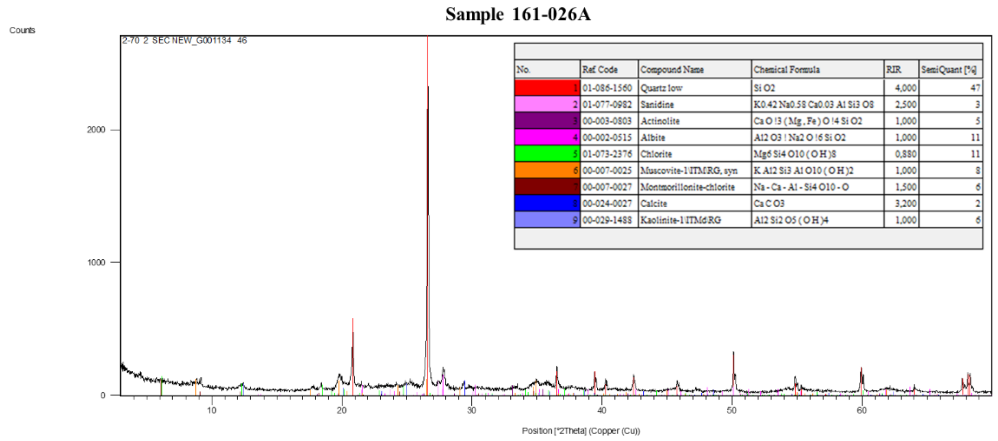

Figure S1: X-ray diffraction (XRD) pattern of sample 161-026A; the legend indicates the semi-quantitative mineralogical composition.

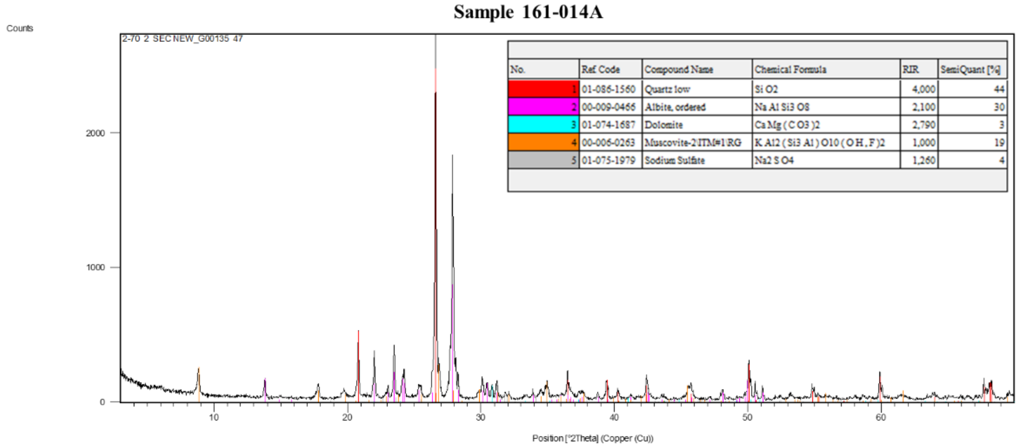

Figure S2: X-ray diffraction (XRD) pattern of sample 161-014A; the legend indicates the semi-quantitative mineralogical composition.

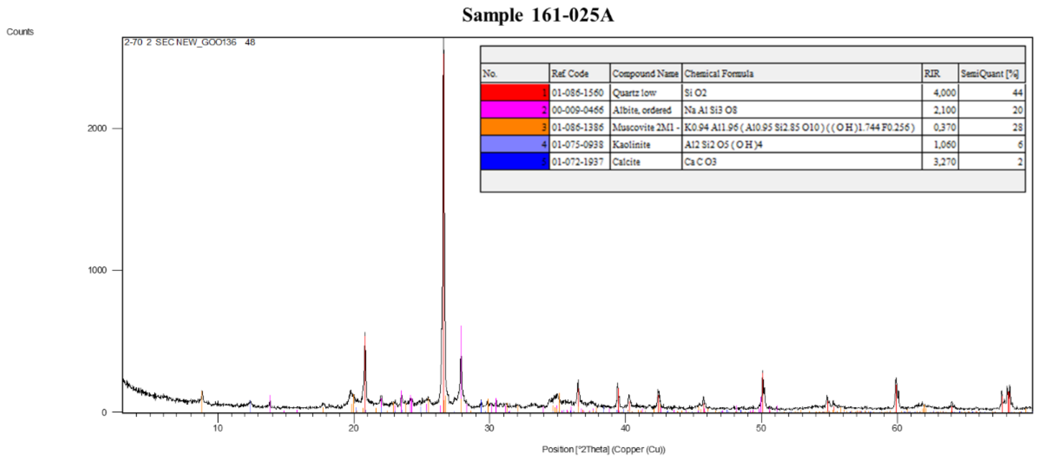

Figure S3: X-ray diffraction (XRD) pattern of sample 161-025A; the legend indicates the semi-quantitative mineralogical composition.

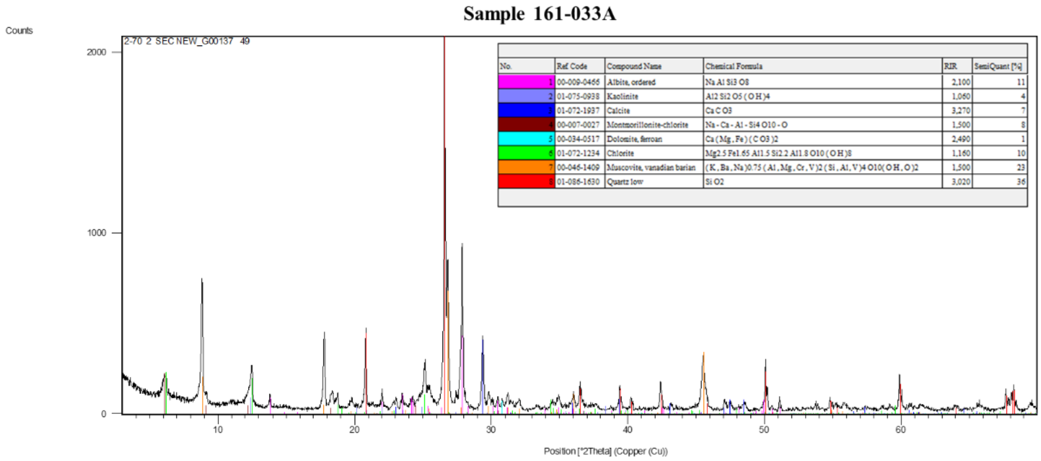

Figure S4: X-ray diffraction (XRD) pattern of sample 161-033A; the legend indicates the semi-quantitative mineralogical composition.

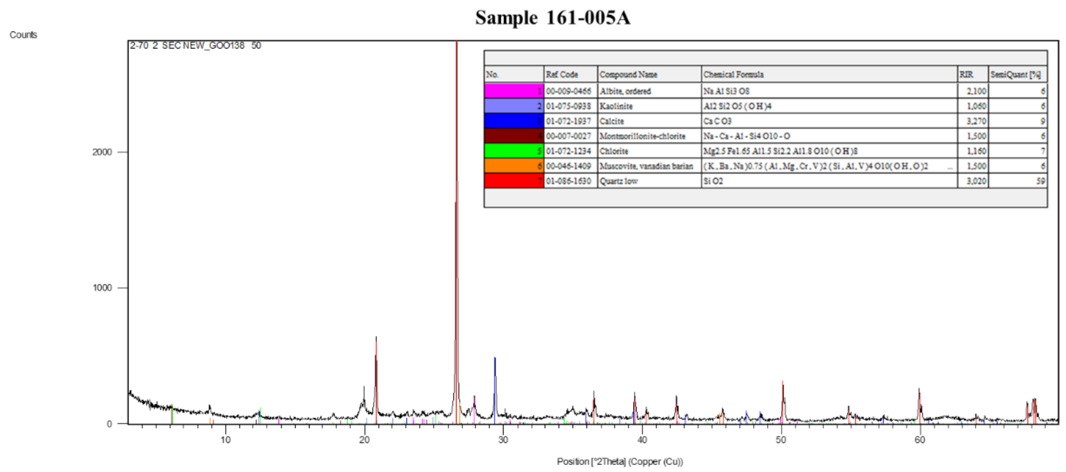

Figure S5: X-ray diffraction (XRD) pattern of sample 161-005A; the legend indicates the semi-quantitative mineralogical composition.

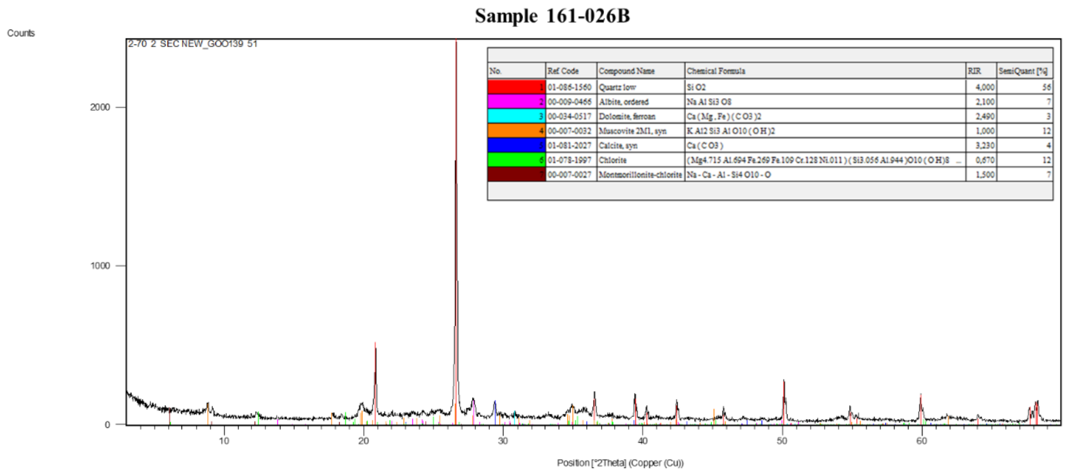

Figure S6: X-ray diffraction (XRD) pattern of sample 161-026B; the legend indicates the semi-quantitative mineralogical composition.

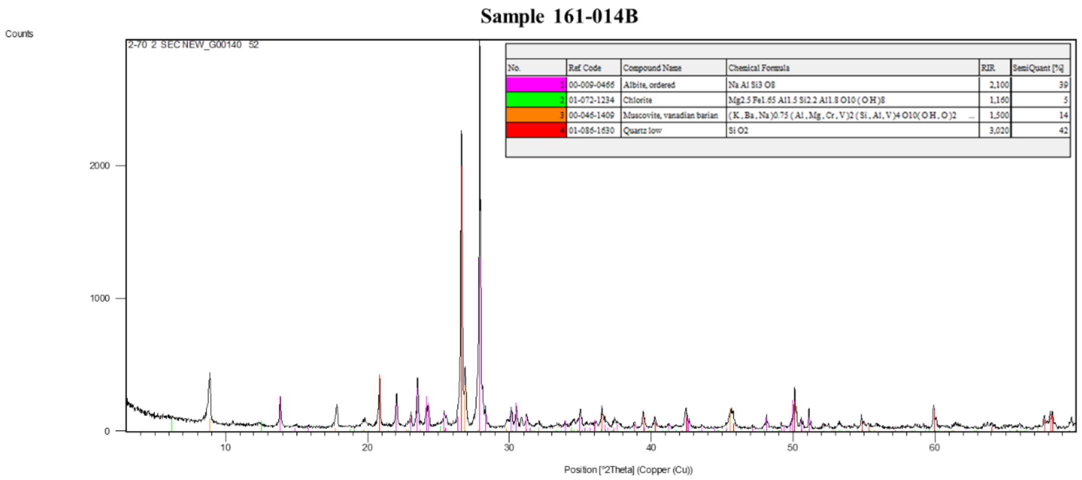

Figure S7: X-ray diffraction (XRD) pattern of sample 161-014B; the legend indicates the semi-quantitative mineralogical composition.

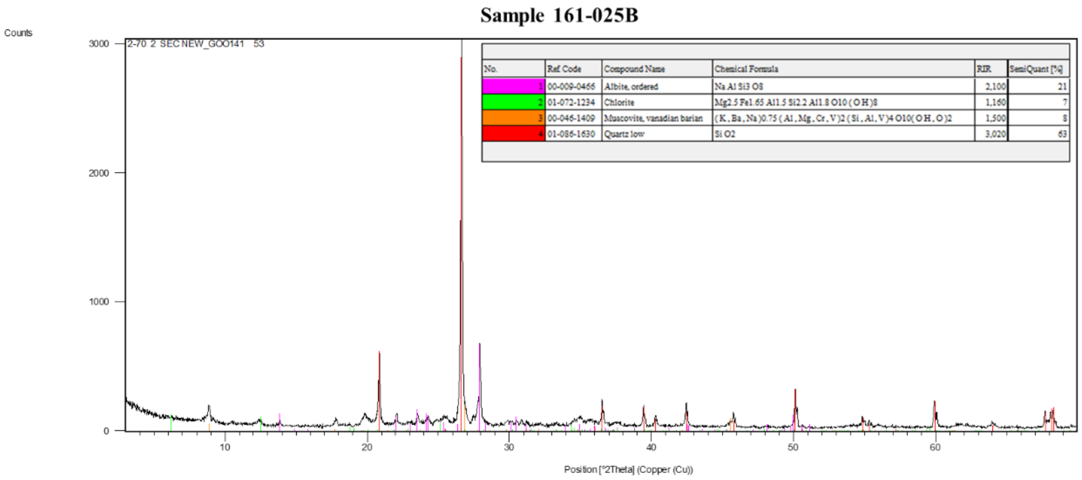

Figure S8: X-ray diffraction (XRD) pattern of sample 161-025B; the legend indicates the semi-quantitative mineralogical composition.

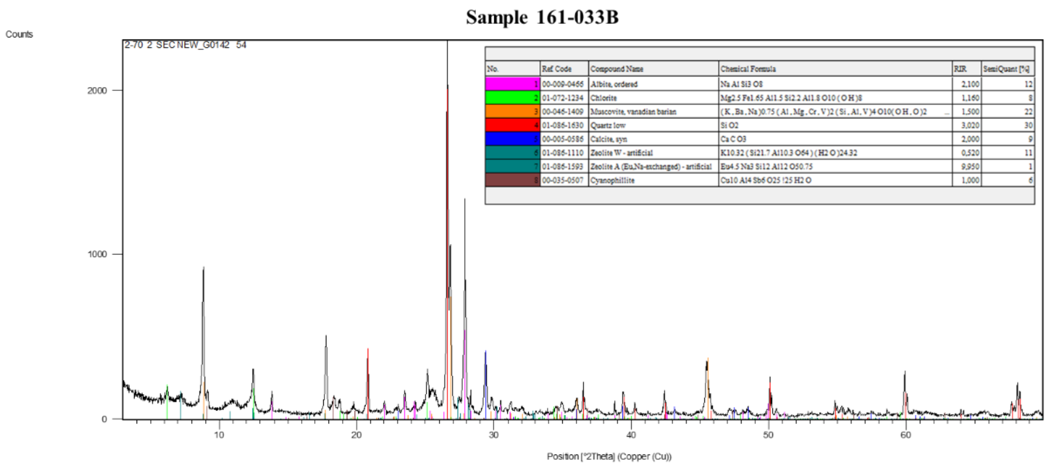

Figure S9: X-ray diffraction (XRD) pattern of sample 161-033B; the legend indicates the semi-quantitative mineralogical composition.

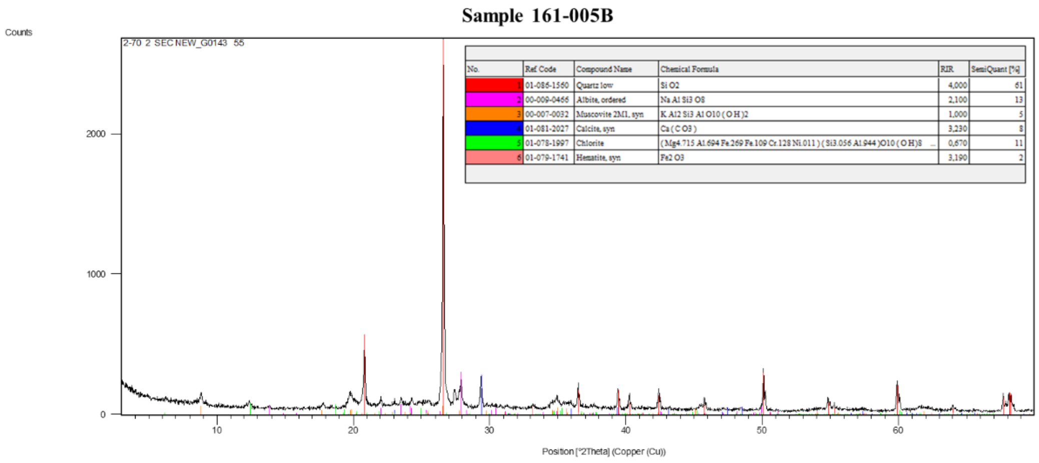

Figure S10: X-ray diffraction (XRD) pattern of sample 161-005B; the legend indicates the semi-quantitative mineralogical composition.
